# Diagnostic accuracy of flat-panel computed tomography in assessing cerebral perfusion in comparison with perfusion computed tomography and perfusion magnetic resonance: a systematic review

**DOI:** 10.1007/s00234-019-02285-y

**Published:** 2019-09-16

**Authors:** Ernst L. Stille, Ilaria Viozzi, Mark ter Laan, Frederick J.A. Meijer, Jurgen J. Futterer, Maroeska M. Rovers

**Affiliations:** 1 Department of Radiology and Nuclear Medicine, Radboud University Medical Center, Geert Grooteplein Zuid 10, 766, PO Box 9101, 6500HB Nijmegen, The Netherlands; 2 Department of Neurosurgery, Radboud University Medical Center, Geert Grooteplein Zuid 10, 725, 6525GA Nijmegen, The Netherlands; 3 Department of Operating Rooms, Radboud University Medical Center, Geert Grooteplein Zuid 10, 715, 6525GA Nijmegen, The Netherlands

**Keywords:** Cerebral perfusion imaging, Flat-panel detector computed tomography, Perfusion computed tomography, Perfusion magnetic resonance

## Abstract

**Purpose:**

Flat-panel computed tomography (FP-CT) is increasingly available in angiographic rooms and hybrid OR’s. Considering its easy access, cerebral imaging using FP-CT is an appealing modality for intra-procedural applications. The purpose of this systematic review is to assess the diagnostic accuracy of FP-CT compared with perfusion computed tomography (CTP) and perfusion magnetic resonance (MRP) in cerebral perfusion imaging.

**Methods:**

We performed a systematic literature search in the Cochrane Library, MEDLINE, Embase, and Web of Science up to June 2019 for studies directly comparing FP-CT with either CTP or MRP in vivo. Methodological quality was assessed using the QUADAS-2 tool. Data on diagnostic accuracy was extracted and pooled if possible.

**Results:**

We found 11 studies comparing FP-CT with CTP and 5 studies comparing FP-CT with MRP. Most articles were pilot or feasibility studies, focusing on scanning and contrast protocols. All patients studied showed signs of cerebrovascular disease. Half of the studies were animal trials. Quality assessment showed unclear to high risks of bias and low concerns regarding applicability. Five studies reported on diagnostic accuracy; FP-CT shows good sensitivity (range 0.84–1.00) and moderate specificity (range 0.63–0.88) in detecting cerebral blood volume (CBV) lesions.

**Conclusions:**

Even though FP-CT provides similar CBV values and reconstructed blood volume maps as CTP in cerebrovascular disease, additional studies are required in order to reliably compare its diagnostic accuracy with cerebral perfusion imaging.

**Electronic supplementary material:**

The online version of this article (10.1007/s00234-019-02285-y) contains supplementary material, which is available to authorized users.

## Introduction

Over the last two decades, flat-panel detector (FD) technology has been introduced into many (neuro-) interventional angiographic suites for diagnostic and therapeutic purposes. Flat-panel detector computed tomography (FP-CT) uses FD technology mounted on a rotational C-arm to generate volumetric imaging with a high spatial resolution [1]. It allows the acquisition of high-quality 3D-vascular images and CT comparable images to study brain parenchyma [1–3].

More recently, FP-CT has been introduced in the hybrid operating room (OR). Given its easy access, FP-CT is an appealing imaging modality for intra-operative applications. In neurosurgery, magnetic resonance (MR) imaging is already used intra-operatively to control the extent of tumor resection in procedures involving craniotomy [4]. The use of cerebral perfusion imaging during these procedures could yield relevant information on the extent of tumor resection, as well as perfusion changes in the area of interest. Using FP-CT for assessing cerebral perfusion could provide a valuable and pragmatic way of intra-operative feedback in surgical procedures, complementing MR. Outside the hybrid OR, cerebral perfusion is commonly assessed with computed tomography (CTP) or magnetic resonance (MRP). Both CTP and MRP have been reported to allow quantitative assessment of (relative) perfusion parameters like cerebral blood volume (CBV) and cerebral blood flow (CBF), showing good agreement with correlation coefficients of up to *r* = 0.95 [5]. Because of its wide accessibility and speed, CTP is part of the diagnostic workup for acute stroke patients at many institutions [6]. However, CTP requires the use of iodinated contrast and exposes patients to radiation.

MRP comprises two contrast-dependent sequences (dynamic susceptibility contrast (DSC), dynamic contrast enhanced (DCE)) and one non-contrast sequence (arterial spin labeling (ASL)). MRP can be applied in evaluating the ischemic penumbra in acute ischemic stroke, selecting patients for reperfusion treatment [7]. MRP is more commonly used for the assessment of perfusion in cerebral gliomas in estimating tumor grade pre-operatively and to distinguish tumor recurrence from radiation necrosis post-treatment [8].

Given the growing availability of FP-CT in the hybrid OR, our aim was to study the diagnostic accuracy of FP-CT regarding cerebral perfusion as compared with the more established techniques of CTP and MRP [9]. Even though FP-CT is already in use to study cerebral perfusion, a systematic review to compare its accuracy with CTP and MRP has never been conducted. We therefore performed a systematic literature review, including all published information on FP-CT imaging compared directly with either CTP or MRP.

## Materials and methods

The review protocol can be accessed at the website of PROSPERO, the International Prospective Register of Systematic Reviews (https://www.crd.york.ac.uk/PROSPERO/). The protocol was registered under number CRD42017048828. We performed this systematic review and meta-analysis according to the Preferred Reporting Items for Systematic reviews and Meta-Analysis (PRISMA) statement [10].

### Search of the literature

We performed a systematic literature search in Medline, Embase, Cochrane, and Web of Science for studies evaluating the accuracy of different perfusion techniques in assessing cerebral hemodynamics in vivo. Our search strings combined “Cerebral circulation” synonyms with synonyms for “Perfusion imaging” and “Flat Panel detector CT” and either “Computed Tomography” or “Magnetic Resonance Imaging” (see Appendix for complete search strategies). Searches were performed from inception up to June 2019. Endnote X8 (Clarivate Analytics, Boston, MA, USA) was used to filter duplicate articles.

### Study selection

We selected studies that compared the diagnostic accuracy of FP-CT directly with either conventional CTP or MRP. We included articles in all languages and contacted the authors of conference abstracts asking for any full-text publications reporting the complete results of their study. Considering the recent introduction of FP-CT, only a limited amount of human studies has been conducted. Therefore, animal studies are included as well but these studies are presented and discussed separately. Studies were excluded if no direct comparison between the two imaging modalities was performed, or if the same patient data had already been published in other articles.

In both CT-based techniques, iodinated contrast was used. We selected MRI studies that used perfusion weighted imaging (PWI), DSC, DCE, or ASL. Selected studies reported on absolute or relative perfusion parameters were used in quantifying cerebral hemodynamics: cerebral blood volume in ml/100 g (CBV), cerebral blood flow in ml/100 g/min (CBF), mean transit time in seconds (MTT), or time to peak in seconds (TTP).

Covidence web-based SR software (Veritas Health Innovation, Melbourne, Australia) was used to assist in the process of screening and selection of abstracts and articles. Two reviewers (ES and IV) independently assessed the eligibility of the identified papers. Any disagreements were resolved by discussion with a third reviewer (MR).

### Data extraction

We extracted data on patient, study, imaging, and contrast characteristics for all studies included. Study population characteristics comprised human or animal (including species), age, whether general anesthesia was given (including used medication) and the pathology explored. Study characteristics included author and year of publication, study design, imaging methods compared, and method of analysis. Imaging characteristics included machines used, number and duration of sweeps indicating static or dynamic imaging, angle of gantry rotation, total amount of images, and radiation dose. Contrast protocol characteristics focus on FP-CT and consist of the concentration and total amount used, route of administration, and X-ray delay. Two reviewers extracted data for all studies using a standardized data extraction form.

### Quality assessment

Both reviewers (ES and IV) independently assessed the risk of bias and concerns regarding applicability to the research question at study level using the validated Quality Assessment of Diagnostic Accuracy Studies (QUADAS-2) tool [11]. Four domains were scored as follows: (1) patient selection, which describes the method for patient selection and the patients included; (2) index test, which describes the test being studied and how it was conducted and interpreted; (3) reference standard, which describes the reference standard test used and how it was conducted and interpreted; and (4) flow and timing, which describe the flow of patient inclusion and exclusion, and the interval between index test and reference standard. Any disagreements were resolved by discussion with a third reviewer (MR).

### Data synthesis and analysis

Data on diagnostic accuracy was extracted and pooled if possible. We gathered qualitative data on sensitivity and specificity and inter- and intra-observer variability in Cohen’s kappa coefficient. Quantitative data on absolute and relative perfusion values was gathered in CBV, CBF, MTT, or TTP. Correlations between techniques are shown using Pearson’s *r* correlation coefficient and agreement between tests is reported in Bland-Altman mean difference test. If extracted data were too heterogeneous to allow for pooling, data will be presented descriptively.

## Results

### Search of the literature

**Fig. 1:**
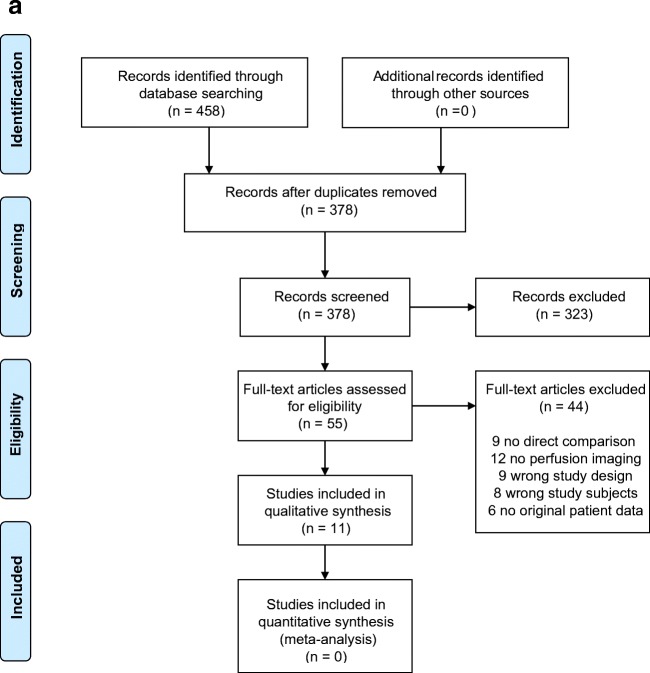

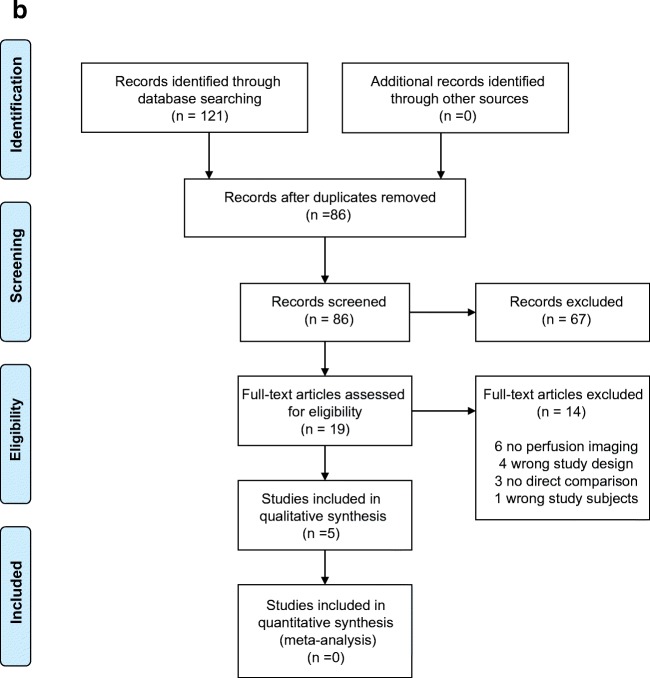
**a** Prisma flowchart FP-CT vs. CTP. **b** Prisma flowchart FP-CT vs. MRP

Our systematic searches for studies that compared FP-CT with CT yielded 458 records and FP-CT with MRI 121 records, which were reduced to 378 and 86 after removing duplicates (see also Fig. 1a and b respectively). Based on title and abstract, 323 and 67 articles were excluded due to incompatibility with our eligibility criteria. Full texts of the remaining 55 and 19 studies were screened and another 44 and 14 articles were excluded, mostly due to a lack of perfusion imaging or no direct comparison between techniques. A total of 11 articles could be included in this systematic review on FP-CT versus CT and 5 articles on FP-CT versus MR.

### Study and patient characteristics

Tables 1 and 2 provide an overview of the included articles and their study design and population for the comparison of FP-CT versus CT and FP-CT versus MR, respectively.

**Table 1:** Study characteristics: FP-CT vs. CTP

	Author	Study characteristics		Population characteristics		
		Design	Population	Diagnosis	Age	
					Mean	Range
Human studies	Fiorella (2014)	Multicenter pilot study	56 patients	CVA	62.9	25–86
	Struffert (2012)	Cohort	16 patients	MCA occlusion, unilateral	69.6	–
	Struffert (2010)	Cohort	25 patients	CVA	58.3	–
	Zhang (2013)	Cohort	12 patients	CVA	52	20–76
	Zhang (2012)	Cohort	20 patients	CVA	55.2	30–70
Animal studies	Ahmed (2009)	Animal trial	12 dogs	Healthy subjects	–	–
	Beuing (2014)	Animal trial	7 sheep	MCA occlusion, unilateral	–	–
	Bley (2010)	Animal trial	20 dogs	MCA occlusion, unilateral	–	–
	Ganguly (2011)	Animal trial	5 pigs	Healthy subjects	–	–
	Royalty (2013)	Animal trial	7 dogs	ICA/MCA occlusion, unilateral	–	–
	Yasuda (2012)	Animal trial	12 dogs	ICA/MCA occlusion, unilateral	–	–

*CVA*, cerebrovascular accident; *MCA*, middle cerebral artery; *ICA*, internal carotid artery

**Table 2:** Study characteristics: FP-CT vs. MRP

	Author	Study characteristics		Population characteristics		
		Design	Population	Diagnosis	Age	
					Mean	Range
Human studies	Garcia (2017)	Cohort	5 patients	Arteriovenous malformation	–	–
	Chen (2018)	Cohort	13 patients	Carotid stenosis > 70%	67.1	45–78
	Kamran (2015)	Cohort	26 patients	DCI after SAH	55	–
	Kuriyama (2018)	Retrospective cohort	10 patients	Unruptured aneurysm	58.1	40–71
	Struffert (2015)	Cohort	12 patients	LVO	72	–

*DCI*, delayed cerebral ischemia; *SAH*, subarachnoid hemorrhage; *LVO*, large vessel occlusion

For the comparison of FP-CT versus CT, four prospective cohort studies [12–15], one multicenter pilot study [16], and six animal studies could be included [17–22]. The number of included participants or animals per study varied between 12 and 56 patients (median 20), and 5 and 12 animals (median 7). All five human studies only included patients with signs of cerebrovascular disease. Although a wide variety of diagnoses were studied (including aneurysms, vasospasms, and dural fistulas), most participants suffered from large vessel occlusion or stenosis. All examinations were done in the (angio-) intervention room and none of the patients was anesthetized. All six animal trials were performed prospectively using large adult mammals, mainly canines and sheep. The animals were either healthy or suffered from cerebrovascular disease, e.g., artificially created occlusions of the middle cerebral artery or the internal carotid artery. All animals were under general anesthesia to reduce motion artifacts.

For the comparison of FP-CT versus MR, four prospective cohort studies [23–26] and one retrospective study [27] could be included. Numbers of included participants per study ranged from 5 to 26 (median 12). All included patients suffered from symptoms of cerebrovascular disease: arteriovenous malformations (*n* = 5) [26], unruptured cerebral aneurysms (*n* = 10) [27], large vessel occlusion (*n* = 12) [23], carotid stenosis (*n* = 13) [25], and delayed cerebral ischemia after subarachnoid hemorrhage (*n* = 26) [24]. All studies were conducted in the (angio-) intervention room and none of the patients was under general anesthesia.

### Risk of bias assessment

**Fig. 2:**
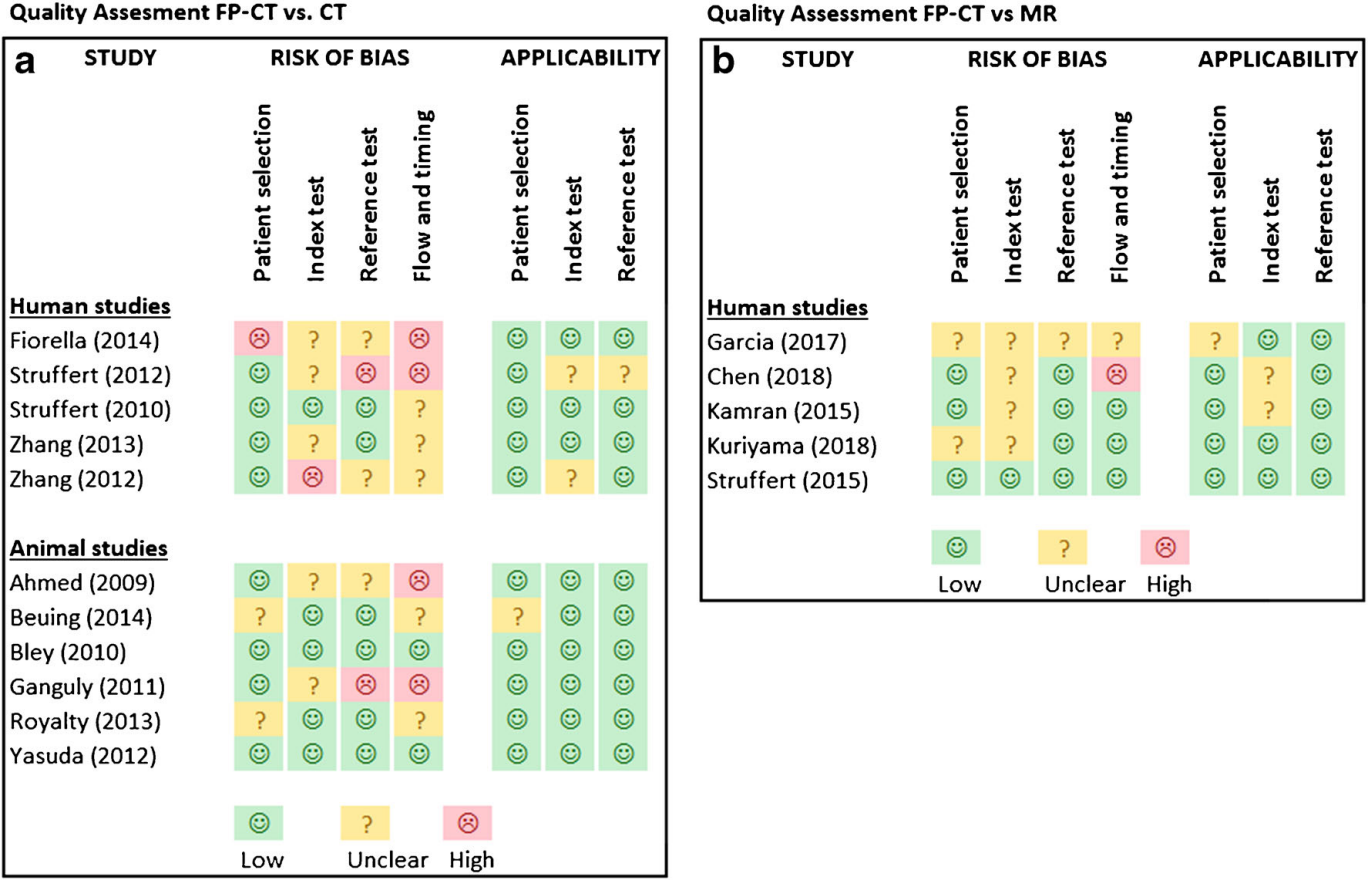
**a** and **b** Quality assessment FP-CT vs. CTP/MRP

Figure 2a and b show the results of the quality assessment. Risk of bias was scored medium to high in four different domains and there was little or low concern regarding applicability to the research question. Overall, the quality of studies was moderate to poor.

### FP-CT vs. CTP

Of the human studies comparing FP-CT versus CT, one study had a high risk of bias regarding the patient selection due to uncertainties in this selection and heterogeneity in the final study population [16]. Regarding the index test, three studies had an unclear risk of bias due to lack of reporting, or the use of different machines [12, 13, 16]. One study was given a high risk of bias because observers were aware of results of the reference standard while interpreting the index test [14]. In the reference standard domain, two studies had an unclear risk of bias and one study had a high risk of bias due to the use of different machines [12, 14, 16]. In the flow and timing domain, three studies had an unclear risk and two studies a high risk of bias [12, 16] due to the use of different machines, inappropriate time between tests, and cases being excluded from the analyses without specification [13–15].

Of the animal studies comparing FP-CT and CT, two studies had an unclear risk of bias in the patient selection domain due to inappropriate exclusions [20, 21]. One of these had concerns regarding applicability as well because of significant differences in (vascular) anatomy and the subsequent need to perform surgery in order to create an artificial stroke. For the index test domain, two studies had an unclear risk of bias because of the use of unspecified thresholds [18, 22]. In the reference standard domain, one study [22] had a high risk of bias due to using different reference standards. In the flow and timing domain, two studies had a high risk of bias due to inappropriate intervals between tests of up to 5 days [18, 22].

### FP-CT vs. MRP

Of the five human studies comparing FP-CT with MR, two studies had an unclear risk of bias or concerns regarding applicability in the domain of patient selection [26, 27]. Four studies had an unclear risk of bias for the index test domain [24–27], two of which also had unclear concerns in applicability due to the interpretation of test results [24, 25]. Two studies had an unclear risk of bias in flow and timing due to inappropriate intervals between tests [25, 26].

### Scanning dose and contrast protocols

Tables 3 and 4 show FP-CT imaging details of all studies. Scan and contrast infusion protocols are described separately for the comparison of FP-CT with CTP and FP-CT with MRP. The most relevant findings are summarized below, with a focus on FP-CT.

**Table 3:** Scan protocol specifics: FP-CT vs. CTP

	Author	Scan protocol				Contrast protocol			
		Machine	Sweeps	Sec/sweep	Static/dynamic	Conc. (mg/ml)	Route	Total	Dilution
Human studies	Fiorella (2014)	Artis Zee	2	8	Static	370	iv	60 ml	–
	Struffert (2012)	Artis dBA/Zeego	2	8	Static	350	iv	60 ml	–
	Struffert (2010)	Artis dBA	2	8	Static	350	iv	80 ml	–
	Zhang (2013)	Artis Zeego	2	8	Static	300	ia-AA	48 ml	50%
	Zhang (2012)	Artis Zee	2	8	Static	–	iv	–	–
Animal studies	Ahmed (2009)	Artis dBA	2	5	Static	300–375	iv	–	–
	Beuing (2014)	Artis Zeego	7	4.43 2.83	Dynamic	400	iv	42 ml	–
	Bley (2010)	Artis dBA	2	5	Static	370	iv	25 ml	–
	Ganguly (2011)	Artis dTA	6	4.3	Dynamic	350	ia	40–50 ml	50–100%
	Royalty (2013)	Artis Zeego	9	2.8	Dynamic	370	iv	28 ml	–
	Yasuda (2012)	Artis Zeego	2	5	Static	370	iv ia-AA ia-CCA ia-VA	25 ml 11–15 ml 4.5–15 ml 3–9.9 ml	100% 15–20% 10–30% 10–30%

*iv*, intravenous; *ia*, intra-arterial; *AA*, aortic arch; *CCA*, common carotid artery; *VA*, vertebral artery

**Table 4:** Scan protocol specifics: FP-CT vs. MRP

	Author	Scan protocol				Contrast protocol		
		Machine	Sweeps	Sec/sweep	Static/dynamic	Conc. (mg/ml)	Route	Total
Human studies	Garcia (2017)	Artis dBA	2	8	Static	370	iv	80 ml
	Chen (2018)	Artis	10	5	Dynamic	370	iv	60 ml
	Kamran (2015)	Artis dBA	2	8	Static	370	iv	80 ml
	Kuriyama (2017)	Artis Zee	2	6	Static	300	ia	Variable
	Struffert (2015)	Artis dBA	9	5	Dynamic	350	iv	60 ml

*iv*, intravenous; *ia*, intra-arterial; *AA*, aortic arch

### FP-CT vs. CTP

All human FP-CT scans were performed on Siemens (Erlangen, Germany) “Artis” models (dBA, dTA, Zee, or Zeego). All five studies performed two C-arm rotations of 8 s each, the first without contrast (mask run) and the second with contrast (fill run). Images are post-processed to produce colored blood volume maps, mainly using commercially available software (syngo X Workplace/Dyna PBV neuro). Four studies used contrast intravenously and one study used a catheter in the aortic arch for intra-arterial contrast administration [13]. All studies using iv contrast administered 60 or 80 ml with concentrations varying from 350 to 370 mg iodine per ml. Zhang et al. administered 48 ml of 300 mg/ml contrast intra-arterially, diluted by 50% [13]. Radiation exposure was reported in either Gray or Sievert, in dose per frame or total dose. Only Fiorella et al. actually measured radiation, averaging 219 mGy for FP-CT [16]. All other studies mentioned 0.36 μGy/frame or 2.3 mSv in total, as reported by manufacturers.

Although the same machines are used in six animal studies, scanning protocols show considerable differences. Static imaging uses only 2 rotations and provides CBV in ml/100 g. Three studies used multiple sweeps varying in duration between 2.8 and 4.43 s, providing flow-based parameters like CBF, MTT, and TTP as well [19, 21, 22]. As rotational speed improves, the number of total acquired images decreases. While 8-s C-arm rotations yielded 400 images, a rotation of 2.8 s allowed for a total of 133 images. Four studies administered contrast intravenously, two studies intra-arterially and one study did both. Contrast load ranged from 25 to 42 ml for intravenous use and from 5 to 50 ml for intra-arterial use. Two studies described and compared several arterial injection protocols with small variations in dilution of contrast (range 10–67%), location (aortic arch, common carotid artery, or vertebral artery), and infusion rate (range 1–6 ml/s). Two studies reported radiation dose, both at 1.2 μGy/projection.

### FP-CT vs. MRP

All five human studies used scanners from the Siemens “Artis” models. Two studies used dynamic imaging with 9 or 10 sweeps of 5 s each, both using 60 ml of undiluted contrast administered intravenously [23, 28]. Two studies reported a protocol of two 8-s sweeps with 80 ml of undiluted iv contrast [24, 26]. One study used two 6-s sweeps and tried several dilutions of intra-arterial contrast. With concentrations ranging from 12 to 28%, a contrast dilution of 20% provided the best images [27]. Four studies reported on radiation dose, all at levels of 0.36 μGy/frame or 4.6 mSv in total.

### Study results

Due to the large heterogeneity of included studies, a meta-analysis could not be performed. We therefore will describe the results of the individual studies. Tables 5 and 6 show quantitative results for all human studies.

**Table 5:** Study results: FP-CT vs. CTP in humans

Author	Population	Methods	Parameter(s)	Results
Diagnostic accuracy				
Fiorella (2014)	56 CVA patients	Detection of PBV deficits: any or > 1/3 of vascular territory	CTP-CBV vs FD-PBV	Sens 100% Spec 63–81%
Zhang (2013)	3 patients: CVA, ICH, Moyamoya	IA contrast dose 70%↓ in visual detection of perf. disorders	IVCT-CBV vs IAFD-CBV	Sens 100% Similar results in all patients
Bland-Altman mean differences				
Struffert (2010)	25 CVA patients	CBV in 6 ROI’s	CTP-CBV vs FD-CBV	− 0.077 ± 0.48 ml/100 g
Zhang (2012)	20 CVA patients	CBV in 6 ROI’s	CTP-CBV vs FDCT-CBV	− 0.25 ± 2.79 ml/100 g
Correlation coefficients				
Struffert (2010)	25 CVA patients	CBV in 6 ROI’s	CTP-CBV vs FD-CBV	*r* = 0.79
Zhang (2012)	20 CVA patients	CBV in 6 ROI’s	CTP-CBV vs FDCT-CBV CTP-rCBV vs FDCT-rCBV	Pearson *r* = 0.68 Pearson *r* = 0.76
Struffert (2012)	16 CVA patients	Post-MCA treatment CBV deficits	CTP-CBV vs FP-CT-CBV	*r* = 0.90

CVA, cerebrovascular accident; *ICH*, intracranial hemorrhage

**Table 6:** Study results: FP-CT vs. MRP in humans

Author	Population	Methods	Parameter(s)	Results
Bland-Altman mean differences				
Kamran (2015)	26 patients: DCI after SAH	Cortical VOI’s Subcortical VOI’s	CCT-rPBV vs weighted MR-rCBF/rCBV	0.015 ± 0.119 ml/100 g 0.043 ± 0.118 ml/100 g
Correlation coefficients				
Chen (2018)	13 patients: carotid stenosis	MCA territory ROI’s	FD-CTP-rCBF vs MRP-rCBF FD-CTP-rMTT vs MRP-rMTT FD-CTP-rTTP vs MRP-rTTP	*r* = 0.73 *r* = 0.42 *r* = 0.58
Kamran (2015)	26 patients: DCI after SAH	Cortical VOI’s Subcortical VOI’s	CCT-rPBV vs weighted MR-rCBF/rCBV	*r* = 0.91 *r* = 0.88
Struffert (2015)	12 CVA patients	Qualitative ASPECTS score	CBV CBF, MTT, and TTP	Pearson *r* = 0.49 Pearson *r* = 0.95–0.98
Garcia (2017)	5 AVM patients	Perinidal perfusion	FPD-CT vs DSC-CBV FPD-CT vs DSC-CBF FPD-CT vs ASL-CBF	Pearson *r* = 0.36 Pearson *r* = 0.47 Pearson *r* = 0.60

*DCI*, delayed cerebral ischemia; *SAH*, subarachnoid hemorrhage; *AVM*, arteriovenous malformation

### FP-CT vs. CTP

For the comparison of FP-CT versus CTP, two human studies reported on diagnostic accuracy. The largest prospective study by Fiorella et al. included 56 patients with ischemic CVA. The study reported a sensitivity and specificity of FP-parenchymal blood volume (PBV) images of 100 and 63% for the detection of CBV deficits larger than one-third of a vascular territory, whereas the sensitivity and specificity to detect any CBV deficit were 100 and 81% [16]. Zhang et al. showed no loss of diagnostic accuracy in 3 out of 12 patients when comparing intra-arterial FP-CT with intravenous CTP in assessing CBV perfusion deficits, while effectively reducing contrast load by 70% [13]. Other articles only compared perfusion parameters and reported on agreement or correlations between (r)CBV values of both techniques. Bland-Altman mean difference analysis was performed in two studies. Both used 6 regions of interest (ROI) per hemisphere, partially corresponding. Results between FP-CTP versus CTP showed mean differences of − 0.077 ± 0.48 ml/100 g in 25 patients and − 0.25 ± −2.79 ml/100 g in 20 patients [14, 15]. Three studies correlated measured perfusion values between FP-CT and CTP. Struffert et al. showed a correlation coefficient of 0.79 in absolute CBV values for 6 ROI’s and Zhang et al. showed moderate correlations in 25 ROI’s, ranging from 0.68 in CBV values to 0.76 in rCBV values [14, 15]. In another study, Struffert et al. correlated CBV deficits measured after recanalization therapy in the angiography suite. Post-treatment FP-CT-CBV deficit volumes showed good correlations with follow-up CT (*r* = 0.9, *p* < 0.05) and multi-slice CT (*r* = 0.9, *p* < 0.05) [12]. Zhang et al. compared the symmetry of contrast distribution in healthy brains of intravenous CTP-rCBV with intra-arterial FP-CT-rCBV and measured similar values in all regions, with total values of 1.01 ± 0.14 for ivCTP and 0.94 ± 0.18 for iaFP-CT (1.0 for perfect symmetry) [13].

Three animal studies compared diagnostic accuracy between techniques. Beuing et al. reported an average sensitivity and specificity of 94% and 83% in the detection of large vessel occlusions in 7 sheep by two observers (Cohen kappa = 0.86) [21]. Bley et al. studied 50 canine CBV maps and compared FP-CT with CTP in detecting MR-DWI confirmed infarctions. Three observers showed average true positive (TP) readings of 84% for FP-CT and 63% for CTP and false negative (FN) readings of 12% for FP-CT and 25% for CTP [17]. Yasuda et al. compared FP-CT with CTP using three different contrast injection protocols and reported similar TP and FN rates from two observers, albeit in a mere 6 animals [19]. Royalty et al. measured interobserver variability in detecting perfusion lesions on 24 CBV and CBF maps, showing a kappa coefficient of 0.74 for CTP and perfect agreement for FP-CT [20]. Other articles mostly performed exploratory analyses regarding the feasibility of FP-CT perfusion measurements, focusing on the development of robust scanning and contrast injection protocols [18, 19, 22].

### FP-CT vs. MRP

For the comparison of FP-CT versus MRP, no studies reported diagnostic accuracy. Chen et al. compared FP-CT and MRP (DSC) in seven different perfusion parameters and showed moderate correlations ranging from 0.42 to 0.73 [25]. Struffert et al. [23] performed a qualitative comparison of FP-CT and MRP using the ASPECTS scoring system for perfusion deficits [29], showing a low Pearson correlation between observers of 0.40–0.49 in CBV and high correlations (0.95–0.98) in CBF, MTT, and TTP [23]. Garcia et al. showed variation coefficients for several techniques among which FP-CT (21.20%), DSC-rCBF (21.30%), and ASL-CBF (11.00%). FP-CT datasets of five patients showed moderate to poor correlations ranging from 0.60 (ASL-CBF) to 0.36 (DSC-CBV) [26]. Kamran et al. compared FP-rPBV with weighted perfusion parameters combining MR-CBV and MR-CBF. Correlation coefficients ranging from 0.72 to 0.91 were reported for cortical ROI’s, with mean differences in Bland-Altman plots as low as 0.015 ± 0.119 ml/100 g. Subcortical ROI’s showed correlations from 0.69 up to 0.88 with a mean difference of 0.043 ± 0.118 ml/100 g [24].

## Discussion

### Summary of evidence

This systematic review includes 11 studies comparing FP-CTP with CTP and 5 studies comparing FP-CTP with MRP. Quality assessment showed low concerns regarding applicability but moderate to high risks of bias, predominantly in the domains of both the index and reference test and the domain of flow and timing. Performing a meta-analysis was not possible due to both the quality of the studies and the large heterogeneity of study populations and designs, the majority being feasibility studies focusing on scanning and contrast protocols. Eleven studies used 2 C-arm sweeps and five studies investigated flow-based dynamic imaging with 6 to 10 gantry sweeps. Most studies (10 human and 4 animal trials) included subjects with cerebrovascular disease and two animal trials studied healthy subjects [18, 30]. Two human studies [13, 16] and three animal studies [17, 19, 21], all comparing FP-CT with CTP, reported on diagnostic accuracy. Although small by design, all five studies reported good to excellent sensitivity and moderate to good specificity for the detection of perfusion deficits in subjects with ischemic stroke. The largest study by Fiorella et al. showed a sensitivity and specificity of 100% and 81% in 56 patients [16], but with high risks of bias. In addition, good correlations [12, 14, 15] and small mean differences [14, 15] have been reported indicating that FP-CT imaging in assessing cerebral perfusion is feasible and provides blood volume images of similar quality as CTP in patients with cerebrovascular disease.

### Strengths and limitations

The strength of our systematic review is that we provide an extensive evaluation of available evidence of FP-CT applications in assessing cerebral perfusion while revealing existing knowledge gaps in current literature.

Some limitations should also be discussed. First, most trials were feasibility or pilot studies showing a large heterogeneity of study population and designs. Imaging and contrast injection protocols were still being developed and fine-tuned, making a meaningful comparison nearly impossible. Only 5 out of 16 articles studied the diagnostic accuracy of the techniques compared, answering our main research question. In addition, risks of bias were high or unclear, making the summarized evidence limited at best.

Second, considering their technical and mathematical background, even directly comparing perfusion parameters like CBV between (FP-)CTP and MRP is not feasible. The relation between contrast-concentration and signal intensity is non-linear in MRI and different mathematical models are used to calculate perfusion parameters. Values can differ significantly between measurements, especially when comparing different techniques. Parameters like blood volume in ml/100 g can suggest (non-existing) calibrated measurements and should be interpreted with caution.

Third, half of the included studies were animal studies. Designs of animal trials in general can introduce selection bias or performance bias [31]. In addition, general anesthesia and differences in vascular anatomy like the presence of a rete mirabile in dogs, sheep, and swines may influence flow and distribution of contrast material [21]. Not necessarily of importance when exploring technical feasibility, these factors do make the transferability from animal to humans unclear.

Lastly, only patients or animals with cerebrovascular disease were studied in the included papers, leaving other applications unexplored. FP-CT scanners are mostly available in angiographic suites where many patients are diagnosed and/or treated, providing plenty of opportunity for clinical studies. In addition, cerebrovascular diseases are particularly suitable for exploring perfusion imaging, as large perfusion deficits are easy to detect. Even though FP-CT scanners are more and more common in hybrid OR’s, evidence for other (intra-operative) applications of FP-CT cerebral perfusion imaging is not available yet.

### Clinical implications

Current literature is of insufficient quality to fully support the use of FP-CT instead of CTP or MRP in cerebral perfusion imaging. Studies so far do indicate comparable preliminary results in specific applications and some recommendations for daily clinical practice can be made. FP-CT provides similar values and reconstructed blood volume maps as CTP in cerebrovascular diseases [12, 32]. Sensitivity for perfusion lesions was high in all studies, but specificity was inferior to CTP and MRP. Multiple studies indicated a slight overestimation of perfusion deficits, which has to be taken into account when using FP-CT. Royalty et al. measured CBV and CBF with both techniques and FP-CT showed values two or three times as high as CTP [20]. Fiorella et al. showed a slight overestimation of CBV lesions with all sensitivities at 100% while specificities dropped to 56% [16]. As clinicians assess perfusion deficits in patients with ischemic stroke, a potential fixed bias and possible outliers must be anticipated in order to prevent false positives.

Some technical considerations can be added as well. In accordance with the assumptions of the indicator dilution theory, intra-arterial administration of a short and concentrated bolus of contrast material can reduce contrast load while maintaining image quality [13, 19, 27]. While most authors used the aortic arch, contrast administered through the vertebral artery does not distribute evenly, making it unfit for whole brain imaging [19]. Depending on administration route, the scanning delay after contrast administration differed from 8 to 19 s in the included studies. Several methods were used to establish the optimal moment to start the scan, number one being maximal opacification of venous structures on fluoroscopic images. Reaching a steady state of contrast with optimal scanning delay times could improve image quality and provide the most reliable results.

High spatial resolution is one of the strengths of FP-CT, as reflected in applications implemented so far [33]. High spatial resolution in cerebral FP-CT imaging enables clear visualization of small vessels and reduces partial volume effects. However, the main technical limitation of present-day FP-CT scanners is low temporal resolution. Temporal sampling > 0.5–3 s can result in unreliable quantification of dynamic perfusion parameters in patients with ischemic stroke [34–37]. The two 8-s sweeps covering ~ 200° used in human studies are insufficient to reliably quantify CBF and MTT [16, 26]. New developments in dynamic imaging with multiple faster sweeps demonstrate the feasibility of flow-based perfusion imaging using FP-CT, although initial results show moderate correlations and qualitative outcomes [23, 25].

One study measured radiation exposure for both techniques and saw similar values of 219 mGy for FP-CT and 204 mGy for CTP [16]. All other studies reported doses per frame of 0.36 μGy/image as indicated by manufacturers. Most imaging protocols reach total effective doses of > 2 mSv per acquisition, which is considerable, especially in procedures requiring repeated imaging and younger patients. Radiation exposure must be closely monitored and should always be proportioned to clinical benefits to ensure safety in future applications, in particular when adding sweeps or images in order to increase temporal resolution.

Since its introduction in the angiographic suite and more recently in the hybrid OR, FP-CT is increasingly used to assess cerebral hemodynamics. While the use of FP-CT seems feasible and provides blood volume images comparable with CTP, additional human studies of adequate size must be conducted to validate the use of FP-CT in assessing cerebral perfusion. Scanning and injection protocols should be standardized, and terminology of techniques and parameters must be used in appropriate and consistent ways. New studies should report on diagnostic accuracy and study populations must be carefully selected with future applications in mind.

## Conclusion

Even though FP-CT provides similar values and reconstructed blood volume maps as CTP in patients with cerebrovascular disease, its diagnostic accuracy remains unclear. Additional methodological sound studies are required in order to reliably determine the diagnostic accuracy of FP-CT in assessing cerebral perfusion.

## Electronic supplementary material

ESM 1 (DOCX 17.0 kb)

## Abbreviation

CTP: Computed tomography perfusion
CVA: Cerebrovascular accident
DCE: Dynamic contrast enhanced
DSC: Dynamic susceptibility contrast
FD: Flat-panel detector
FP-CT: Flat-panel computed tomography
MRP: Magnetic resonance perfusion
OR: Operating room
