# Supplementary material for: Diagnostic accuracy of flat-panel computed tomography in assessing cerebral perfusion in comparison with perfusion computed tomography and perfusion magnetic resonance: a systematic review
Source: Neuroradiology. 2019 Sep 16;61(12):1457–68. doi: 10.1007/s00234-019-02285-y (PMC6848034; doi:10.1007/s00234-019-02285-y)
Supplement: Supplementary file 1 — (DOCX 17.0 kb) [file 234_2019_2285_MOESM1_ESM.docx]

FP-CT vs CTP (458)

**Medline (124)**

("Cerebrovascular Circulation"[Mesh] OR cerebral[tiab] OR cerebrovascular[tiab] OR

brain[tiab] OR ICA[tiab] OR internal carotid artery[tiab] OR internal carotid arteries[tiab] OR

MCA[tiab] OR Middle cerebral artery[tiab] OR Middle cerebral arteries[tiab] OR vertebral

artery[tiab] OR vertebral arteries[tiab] OR basilar artery[tiab]) AND ("Cone-Beam Computed

Tomography"[Mesh] OR CBCT[tiab] OR CB CT[tiab] OR Cone beam [tiab] OR fdct[tiab] OR fdct[tiab] OR flatpanel[tiab] OR flat panel[tiab] OR flat detector [tiab] OR flat detector [tiab] OR C-arm[tiab] OR Volumetric CT[tiab] OR Volumetric computed tomography[tiab] OR Volume CT[tiab] OR Volume Computed Tomography[tiab]) AND ("Perfusion Imaging"[Mesh] OR "Perfusion"[Mesh] OR "Regional Blood Flow"[Mesh] OR blood supply[subheading] OR

perfusion[tiab] OR hemodynamics[tiab] OR CBV[tiab] OR blood volume[tiab] OR CBF[tiab] OR

blood flow[tiab] OR cerebral flow[tiab] OR flow measurement*[tiab] OR PBV[tiab] OR

MTT[tiab] OR mean transit time[tiab]) AND (“Tomography, X-Ray Computed”[Mesh] OR

Computed tomography[tiab] OR Computer assisted tomography[tiab] OR CT[tiab] OR

CTP[tiab] OR CAT[tiab] OR MDCT[tiab] OR 4DCT[tiab])

**Embase (189)**(Exp Brain circulation/ OR Exp Brain blood flow/ OR Brain perfusion/ OR ((cerebral OR cerebrovascular OR brain OR ICA OR internal carotid artery OR internal carotid arteries OR MCA OR Middle cerebral artery OR Middle cerebral arteries OR vertebral artery OR vertebral arteries OR basilar artery).ti,ab,kw.)) AND (Cone beam computed tomography/ OR ((CBCT OR CB CT OR Cone beam OR fdct OR fd-ct OR flatpanel OR flat panel OR flatdetector OR flat detector OR C-arm OR Volumetric CT OR Volumetric computed tomography OR Volume CT OR Volume Computed Tomography).ti,ab,kw.)) AND (exp blood flow/ OR perfusion/ OR hemoperfusion/ OR exp organ perfusion/ OR tissue perfusion/ OR ((perfusion OR hemodynamics OR CBV OR blood volume OR CBF OR blood flow OR cerebral flow OR flow measurement* OR PBV OR MTT OR mean transit time).ti,ab,kw.)) AND (computer assisted tomography/ OR computed tomographic angiography/ OR four dimensional computed tomography/ OR high resolution computer tomography/ OR multidetector computed tomography/ OR spiral computer assisted tomography/ OR whole body ct/ OR ((Computed tomography OR Computer assisted tomography OR CT OR CTP OR CAT OR MDCT OR 4DCT).ti,ab,kw.))

**Web of Science (139)**

(cerebral OR cerebrovascular OR brain OR ICA OR “internal carotid artery” OR “internal carotid arteries” OR MCA OR “Middle cerebral artery” OR “Middle cerebral arteries” OR “vertebral artery” OR “vertebral arteries” OR “basilar artery”) AND (CBCT OR “CB CT” OR “Cone beam” OR fdct OR “fd-ct” OR flatpanel OR “flat panel” OR flatdetector OR “flat detector” OR “C-arm” OR “Volumetric CT” OR “Volumetric computed tomography” OR “Volume CT” OR “Volume Computed Tomography”) AND (perfusion OR hemodynamics OR CBV OR “blood volume” OR CBF OR “blood flow” OR “cerebral flow” OR “flow measurement*” OR PBV OR MTT OR “mean transit time”) AND (“Computed tomography” OR “Computer assisted tomography” OR CT OR CTP OR “CT perfusion” OR “CT-Perfusion” OR MDCT OR “MD CT” OR “multidetector computed tomography” OR “multi detector computed tomography” OR 4DCT OR “4D CT”)

**Cochrane (6)**

"Cerebrovascular Circulation"[Mesh] OR "Cerebral":ti,ab OR "cerebrovascular":ti,ab OR "brain":ti,ab OR "ICA":ti,ab OR "internal carotid artery":ti,ab OR "internal carotid arteries":ti,ab OR "MCA":ti,ab OR "Middle cerebral artery":ti,ab OR "Middle cerebral arteries":ti,ab OR "vertebral artery":ti,ab OR "vertebral arteries":ti,ab OR "basilar artery":ti,ab

"Cone-Beam Computed Tomography"[Mesh] OR "CBCT":ti,ab OR "CB CT":ti,ab OR "Cone

beam":ti,ab OR "fdct":ti,ab OR "fd-ct":ti,ab OR "flatpanel":ti,ab OR "flat panel":ti,ab OR "flat

detector":ti,ab OR "flat detector":ti,ab OR "C-arm":ti,ab OR "Volumetric CT":ti,ab OR

"Volumetric computed tomography":ti,ab OR "Volume CT":ti,ab OR "Volume Computed

Tomography":ti,ab

"Perfusion Imaging"[Mesh] OR "Perfusion"[Mesh] OR "Regional Blood Flow"[Mesh] OR blood

supply[subheading] OR "perfusion":ti,ab OR "hemodynamics":ti,ab OR "CBV":ti,ab OR "blood

volume":ti,ab OR "CBF":ti,ab OR "blood flow":ti,ab OR "cerebral flow":ti,ab OR "flow

measurement*":ti,ab OR "PBV":ti,ab OR "MTT":ti,ab OR "mean transit time":ti,ab

“Tomography, X-Ray Computed”[Mesh] OR "Computed tomography":ti,ab OR "Computer

assisted tomography":ti,ab OR "CT":ti,ab OR "CTP":ti,ab OR "CAT":ti,ab OR "MDCT":ti,ab OR

"4DCT":ti,ab

FP-CT vs MRP (121)

**Medline (32)**

("Cerebrovascular Circulation"[Mesh] OR cerebral[tiab] OR cerebrovascular[tiab] OR brain[tiab] OR ICA[tiab] OR internal carotid artery[tiab] OR internal carotid arteries[tiab] OR MCA[tiab] OR Middle cerebral artery[tiab] OR Middle cerebral arteries[tiab] OR vertebral artery[tiab] OR vertebral arteries[tiab] OR basilar artery[tiab]) AND ("Cone-Beam Computed Tomography"[Mesh] OR CBCT[tiab] OR CB CT[tiab] OR Cone beam [tiab] OR fdct[tiab] OR fdct[tiab] OR flatpanel[tiab] OR flat panel[tiab] OR flat detector [tiab] OR flat detector [tiab] OR C-arm[tiab] OR Volumetric CT[tiab] OR Volumetric computed tomography[tiab] OR Volume CT[tiab] OR Volume Computed Tomography[tiab]) AND ("Perfusion Imaging"[Mesh] OR "Perfusion"[Mesh] OR "Regional Blood Flow"[Mesh] OR blood supply[subheading] OR perfusion[tiab] OR hemodynamics[tiab] OR CBV[tiab] OR blood volume[tiab] OR CBF[tiab] OR blood flow[tiab] OR cerebral flow[tiab] OR flow measurement*[tiab] OR PBV[tiab] OR MTT[tiab] OR mean transit time[tiab]) AND (“Magnetic Resonance Imaging”[Mesh] OR Magnetic Resonance Imaging[tiab] OR MR[tiab] OR MRI[tiab] OR Magnetic resonance perfusion[tiab] OR MRP[tiab] OR Fmri[tiab] OR BOLD[tiab] OR blood oxygenated level dependent[tiab] OR DSC[tiab] OR dynamic susceptibility contrast[tiab] OR DCE[tiab] OR dynamic contrast enhanced[tiab] OR ASL[tiab] OR arterial spin labeling[tiab])

**Embase (73)**

(Exp Brain circulation/ OR Exp Brain blood flow/ OR Brain perfusion/ OR ((cerebral OR cerebrovascular OR brain OR ICA OR internal carotid artery OR internal carotid arteries OR MCA OR Middle cerebral artery OR Middle cerebral arteries OR vertebral artery OR vertebral arteries OR basilar artery).ti,ab,kw.)) AND (Cone beam computed tomography/ OR ((CBCT OR CB CT OR Cone beam OR fdct OR fd-ct OR flatpanel OR flat panel OR flatdetector OR flat detector OR C-arm OR Volumetric CT OR Volumetric computed tomography OR Volume CT OR Volume Computed Tomography).ti,ab,kw.)) AND (exp blood flow/ OR perfusion/ OR hemoperfusion/ OR exp organ perfusion/ OR tissue perfusion/ OR ((perfusion OR hemodynamics OR CBV OR blood volume OR CBF OR blood flow OR cerebral flow OR flow measurement* OR PBV OR MTT OR mean transit time).ti,ab,kw.)) AND (nuclear magnetic resonance imaging/ OR cardiovascular magnetic resonance/ OR diffusion weighted imaging/ OR echo planar imaging/ OR functional magnetic resonance imaging/ OR multiparametric magnetic resonance imaging/ OR perfusion weighted imaging/ OR susceptibility weighted imaging/ OR whole body mri/ OR ((Magnetic Resonance Imaging OR MR OR MRI OR Magnetic resonance perfusion OR MRP OR Fmri OR BOLD OR blood oxygenated level dependent OR DSC OR dynamic susceptibility contrast OR DCE OR dynamic contrast enhanced OR ASL OR arterial spin labeling).ti,ab,kw.))

**Web of Science (14)**

(cerebral OR cerebrovascular OR brain OR ICA OR “internal carotid artery” OR “internal carotid arteries” OR MCA OR “Middle cerebral artery” OR “Middle cerebral arteries” OR “vertebral artery” OR “vertebral arteries” OR “basilar artery”) AND (CBCT OR “CB CT” OR “Cone beam” OR fdct OR “fd-ct” OR flatpanel OR “flat panel” OR flatdetector OR “flat detector” OR “C-arm” OR “Volumetric CT” OR “Volumetric computed tomography” OR “Volume CT” OR “Volume Computed Tomography”) AND (perfusion OR hemodynamics OR CBV OR “blood volume” OR CBF OR “blood flow” OR “cerebral flow” OR “flow measurement*” OR PBV OR MTT OR “mean transit time”) AND (“MR perfusion” OR MRP OR BOLD OR “blood oxygenated level dependent” OR DSC OR “dynamic susceptibility contrast” OR DCE OR “dynamic contrast enhanced” OR “dynamic contrast-enhanced” OR ASL OR “arterial spin labeling”)

**Cochrane (2)**

"Cerebrovascular Circulation"[Mesh] OR "Cerebral":ti,ab OR "cerebrovascular":ti,ab OR

"brain":ti,ab OR "ICA":ti,ab OR "internal carotid artery":ti,ab OR "internal carotid

arteries":ti,ab OR "MCA":ti,ab OR "Middle cerebral artery":ti,ab OR "Middle cerebral

arteries":ti,ab OR "vertebral artery":ti,ab OR "vertebral arteries":ti,ab OR "basilar

artery":ti,ab

"Cone-Beam Computed Tomography"[Mesh] OR "CBCT":ti,ab OR "CB CT":ti,ab OR "Cone

beam":ti,ab OR "fdct":ti,ab OR "fd-ct":ti,ab OR "flatpanel":ti,ab OR "flat panel":ti,ab OR "flat

detector":ti,ab OR "flat detector":ti,ab OR "C-armikea ":ti,ab OR "Volumetric CT":ti,ab OR

"Volumetric computed tomography":ti,ab OR "Volume CT":ti,ab OR "Volume Computed

Tomography":ti,ab

"Perfusion Imaging"[Mesh] OR "Perfusion"[Mesh] OR "Regional Blood Flow"[Mesh] OR blood

supply[subheading] OR "perfusion":ti,ab OR "hemodynamics":ti,ab OR "CBV":ti,ab OR "blood

volume":ti,ab OR "CBF":ti,ab OR "blood flow":ti,ab OR "cerebral flow":ti,ab OR "flow

measurement*":ti,ab OR "PBV":ti,ab OR "MTT":ti,ab OR "mean transit time":ti,ab

“Magnetic Resonance Imaging”[Mesh] OR "Magnetic Resonance Imaging”:ti,ab OR "MR":ti,ab OR "MRI":ti,ab OR "Magnetic resonance perfusion":ti,ab OR "MRP":ti,ab OR "FMRI":ti,ab OR "BOLD":ti,ab OR "blood oxygenated level dependent":ti,ab OR "DSC":ti,ab OR "dynamic susceptibility contrast":ti,ab OR "DCE":ti,ab OR "dynamic contrast enhanced":ti,ab OR "ASL":ti,ab OR "arterial spin labeling":ti,ab
